# Seasonal Captures of *Trissolcus japonicus* (Ashmead) (Hymenoptera: Scelionidae) and the Effects of Habitat Type and Tree Species on Detection Frequency

**DOI:** 10.3390/insects12020118

**Published:** 2021-01-29

**Authors:** Nicole F. Quinn, Elijah J. Talamas, Tracy C. Leskey, J. Christopher Bergh

**Affiliations:** 1 Alson H. Smith Jr. Agricultural Research and Extension Center, Virginia Tech, Winchester, VA 22602, USA; cbergh@vt.edu; 2 Florida Department of Agriculture and Consumer Services, Division of Plant Industry, Gainesville, FL 32608, USA; Elijah.Talamas@fdacs.gov; 3 Appalachian Fruit Research Station, USDA ARS, Kearneysville, WV 25430, USA; tracy.leskey@usda.gov

**Keywords:** biological control, parasitoid, *Halyomorpha halys*, *Ailanthus altissima*

## Abstract

**Simple Summary:**

*Trissolcus japonicus*, an important natural enemy of brown marmorated stink bug in Asia, was first detected in the USA in 2014. To investigate when and where *T. japonicus* is found in the field, yellow sticky traps were deployed in the canopy of tree of heaven growing at the edge of small isolated patches, windbreaks, and woodlots in 2018 and 2019. In both years, captures occurred from May to September, with peaks in July and August. Captures of *T. japonicus* were recorded from all three habitats but were not consistently associated with a particular habit. In 2017 and 2018, *T. japonicus* captures were compared between tree of heaven paired with several other *H. halys* host trees growing at the woods edge, and in 2019, captures in tree of heaven, black walnut, and black locust growing in the same windbreaks were compared. *Trissolcus japonicus* and several native *H. halys* parasitoids were captured in all hosts, but there was not a consistent effect of host tree species on *T. japonicus* captures. These results can be used to inform and optimize future surveillance efforts for detecting *T. japonicus* as it continues to expand its range in the USA.

**Abstract:**

*Trissolcus japonicus*, an important egg parasitoid of *Halyomorpha halys* in Asia, was first detected in the USA in 2014. To evaluate the effect of habitat and the seasonality of *T. japonicus* detections in the USA, yellow sticky traps were placed in the canopy of *Ailanthus altissima* growing at the edge of isolated patches of trees, windbreaks, and woodlots in northern Virginia in 2018 and 2019. In both years, captures occurred from May to September, and peaked in July and August. While *T. japonicus* was detected in all habitats, there was not a consistent effect of habitat type on capture frequency. To evaluate tree species effects on *T. japonicus* captures, in 2017 and 2018, yellow sticky traps deployed in the canopy of *A. altissima* bordering apple orchards were paired with a nearby trap in one of several wild tree species along a common woods edge. In 2019, these traps were deployed in *A. altissima*, black walnut, and black locust growing in the same windbreaks. No consistent association between captures of *T. japonicus* or native parasitoids of *H. halys* and the tree species sampled was observed among years. Results are discussed in relation to the ecology and sampling optimization of *T. japonicus*.

## 1. Introduction

*Halyomorpha halys* (Stål) (Hemiptera: Pentatomidae), is a polyphagous invasive stink bug from Asia that has been a severe agricultural and nuisance pest in many parts of the USA since the late 2000s [1]. A widespread outbreak in 2010 resulted in major losses to the apple and peach crops in the Mid-Atlantic USA [2]. To manage *H. halys*, many American tree fruit producers increased their use of broad-spectrum insecticides [3], but this is certainly not considered a sustainable, long-term solution. Ultimately, biological control may play an important role in sustainable *H. halys* management [1]. Surveys of native *H. halys* parasitoids in North America [4] revealed that the species detected most commonly from sentinel egg masses included members of the genera *Anastatus* Motschulsky (Hymenoptera: Eupelmidae), *Telenomus* Haliday (Hymenoptera: Scelionidae), and *Trissolcus* Ashmead (Hymenoptera: Scelionidae). However, field studies have suggested that endemic parasitoids and predators in the USA are not yet regulating *H. halys* populations adequately [4,5].

Detections of North American *H. halys* parasitoids have been somewhat habitat dependent [4]. *Trissolcus* species tended to be more prevalent in ornamental, semi-natural/urban, and forest habitats than in other systems [4], and Herlihy et al. [6] found that this genus predominated in wooded habitats. Parasitization of sentinel *H. halys* eggs did not differ between exotic or native host plants but was greatest at the edge of unmanaged woods [7]. *Anastatus* species were among the most common parasitoids in ornamental systems [8].

*Trissolcus japonicus* (Ashmead) (Hymenoptera: Scelionidae) is one of the most important natural enemies of *H. halys* in Asia [9,10]. The discovery of an adventive population of *T. japonicus* in Maryland in 2014 [11] prompted more intensive and extensive surveillance for *H. halys* parasitoids in North America and Europe, resulting in *T. japonicus* detections in 13 states, Washington, DC [6,12,13,14], Canada, Italy, and Switzerland [15,16,17,18]. Although Abram et al. [19] noted that the effects of egg parasitoids, including *T. japonicus*, on *H. halys* populations have not been quantified, intensified sampling for *T. japonicus* in the USA to track changes in its spread and abundance is warranted and would benefit from a better understanding of its temporal and spatial distributions.

Zhang et al. [10] documented the seasonal parasitism of *H. halys* eggs by *T. japonicus* in Beijing, China, although its seasonal phenology elsewhere has not been reported. In the USA, it has been detected in woodland [6,20,21] and lightly wooded residential habitats [13], and Herlihy et al. [6] reported that it parasitized significantly more *H. halys* sentinel eggs in wooded habitats than in soybean or apple plantings. In both the USA [20] and China [10], *T. japonicus* has also been detected in peach orchards. However, there have not been systematic comparisons of *T. japonicus* detection frequency and relative abundance among the wooded habitats in which it has been found, particularly those adjacent to crops at risk from *H. halys* attack. Moreover, although *T. japonicus* detections in the USA have been most common in arboreal habitats [6,11,21,22,23], its presence and abundance among different *H. halys* tree hosts has not been examined.

Optimizing the efficiency of sampling to track changes in the range and abundance of *T. japonicus* in the USA can be achieved and informed by a greater understanding of potential habitat and plant species effects on its detection frequency, and by documenting its seasonal phenology. Here, studies conducted in northern Virginia, USA, where *T. japonicus* has been present since at least 2015 [21], address adventive *T. japonicus* captures in relation to habitat type, plant species, and seasonality. 

## 2. Materials and Methods

### 2.1. Seasonal Phenology and Habitat Type 

Study sites were unmanaged, wooded habitats adjacent to commercial and experimental orchards in the counties of Frederick (14 sites) and Shenandoah (1 site), Virginia, USA, where *T. japonicus* has been detected consistently since 2015. All sites were within 10 km of Virginia Tech’s Alson H. Smith Jr. Agricultural Research and Extension Center (AHSAREC) (39.112867, −78.284029) in Frederick county, and the same sites were used for trapping in 2018 and 2019. 

Tree of heaven, *Ailanthus altissima* (Mill.) Swingle, is an invasive Asian species [24] that often grows prolifically in disturbed or semi-disturbed locations in Virginia and elsewhere in the USA [25]. In this area, it was the most abundant deciduous tree species at the edge of woodlands bordering tree fruit orchards [26] and supports all *H. halys* life stages [27]. In addition, inspection of the foliage of felled tree of heaven yielded *H. halys* egg masses parasitized by *T. japonicus* [20]. The prevalence of this tree and the occurrence of both *H. halys* and *T. japonicus* on it led to the selection of female tree of heaven for standardized sampling. 

**Figure 1:**
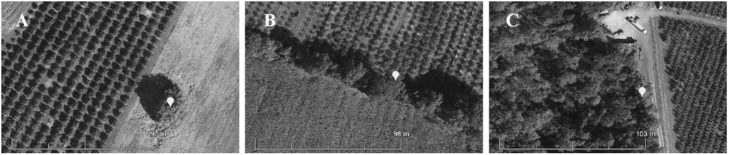
Representative habitat types in which *Trissolcus japonicus* was sampled using yellow sticky traps in the mid-canopy of mature female tree of heaven near orchards in Frederick and Shenandoah counties, VA in 2018 and 2019: (**A**) Isolated patches of predominantly tree of heaven, (**B**) Long, narrow windbreak of mixed wild trees and shrubs, (**C**) Contiguous, unmanaged woodland with wild trees and shrubs. Marker indicates trap location in trees of at the edge of each habitat.

In this area, three common habitat types in which tree of heaven grows are: (1) spatially isolated, often roughly circular patches typically associated with rock breaks in otherwise cultivated or fallow fields; (2) windbreaks or hedgerows; (3) the edge of woodlands (Figure 1). All of these habitat types are commonly associated with commercial tree fruit orchards and thus were selected for sampling (*n* = 5 sites per type). The mean (± SE) distance (km) between isolated patch, windbreak, and woodlot sites was, 3.8 ± 0.5, 6.5 ± 0.7, and 4.2 ± 0.5, respectively, and the mean (± SE) distance (km) between a given site and the site nearest to it was 0.84 ± 0.24. The mean (± SE) area (m^2^) comprised of trees at these sites was; (1) isolated patches 278.6 ± 88.1 (17.3 ± 3.6 m at widest point), (2) windbreaks 2629.2 ± 827.5 (10.7 ± 1.7 m at widest point), and (3) woods 649,866.4 ± 254,141.6 (977.8 ± 130.0 m at widest point). 

As described in Quinn et al. [22], backfolding yellow sticky traps (23 × 28 cm, Alpha Scents, West Linn, OR, USA) deployed atop 4.8 m bamboo poles were used for sampling. Holes were punched through both halves of folded traps, at about 2.54 cm on either side of the center point and about 1.9 cm from the edge. A 45.7 cm length of twist tie was inserted through the holes, the top of the pole was inserted between the trap sides, and the twist tie was used to secure the trap by wrapping and twisting it around the pole and finally to the shank of a wire hook affixed to the pole below the trap. Interlocking tabs on the trap corners ensured that both sides were closely appressed to the pole when deployed. One trap was deployed in the mid-canopy of a mature female tree of heaven growing at the habitat edge at each site by suspending the pole from a lateral branch via the wire hook. Traps were deployed on 3 May and 20 April in 2018 and 2019, respectively, and replaced at 7 ± 2-day intervals through 21 or 30 September in the respective years. 

### 2.2. Host Plant Comparisons

Mature trees at the edge of woodlands (2017 and 2018) and windbreaks (2019) adjacent to tree fruit orchards within 10 km of the AHSAREC were used for trapping. The height and diameter at breast height (DBH) of each sample tree was recorded using a Nikon Forestry Pro Hypsometer (Nikon Corporation, Tokyo, Japan) and measuring tape, respectively. As in the previous study, female tree of heaven (11.2 ± 1.3 m tall, 0.2 ± 0.02 m DBH) was the standard species used in all paired host comparisons. The endemic species used were black walnut, *Juglans nigra*, L. (Fagales: Juglandaceae) (12.4 ± 1.5 m tall, 1.3 ± 0.8 m DBH), black locust, *Robinia pseudoacacia* L. (Fabales: Fabaceae) (8.5 ± 0.9 m tall, 0.13 ± 0.03 m DBH), hackberry, *Celtis occidentalis* L. (Rosales: Cannabaceae) (16.1 ± 5.5 m tall, 0.3 ± 0.06 m DBH), and black cherry *Prunus serotina* Ehrh. (Rosales: Rosaceae) (8.2 m ± 3.7 tall, 0.2 m ± 0.01 DBH), all of which were also among the most common wild trees recorded in this region [26] and are known hosts of *H. halys* [27]. In addition to representing a diversity of plant families, these trees differ in leaf structure (i.e., simple versus complex). The same tree of heaven, black walnut, black locust, and hackberry trees were used in 2017 and 2018, and black cherry was added in 2018. 

Backfolding yellow sticky traps were deployed as described previously. At each site, a trap in female tree of heaven was paired with a trap in one of the aforementioned species (*n* = 5 per species pairing). Trees within pairs were 10 to 25 m apart and the mean distance between pairs was 3.4 ± 0.2 km. Traps were replaced at 7 ± 2-day intervals from 31 July until 29 August 2017 and 13 June until 20 September 2018. 

In 2019, sampling was conducted at five windbreaks, separated by 5.6 ± 0.7 km. At each site, a single yellow sticky trap was deployed as described previously in one tree of heaven (8.5 ± 1.0 m tall, 0.1 ± 0.01 m DBH), one black walnut (10.1 ± 1.02 m tall, 0.4 ± 0.01 m DBH), and one black locust (7.1 ± 1.6 m tall, 0.1 ± 0.0.2 m DBH). Adjacent sampling trees were 23.7 ± 8.6 m apart and the distance between trees at the ends of the sampling area was 47.4 ± 15.9 m. Traps were replaced at 7 ± 1-day intervals from 17 June until 11 August.

### 2.3. Parasitoid Identification

For all studies, all parasitoids of interest captured (i.e., those considered to be potential *H. halys* parasitoids) were tentatively identified in situ in the laboratory, following Talamas et al. [28]. With the exception of *Anastatus* spp., all specimens were sent in situ on a small piece of the trap to E.J. Talamas for species confirmation. Male and female *T. japonicus* captured in the 2018 and 2019 habitat type study were differentiated based on antennal morphology [29]. 

### 2.4. Statistical Analysis

For each year, seasonal detections of *T. japonicus* are presented as total male and female *T. japonicus* from pooled captures across all habitat types by week. To compare *T. japonicus* captures among habitat types, data from each year were pooled across sample dates and analyzed using the Kruskal-Wallis test followed by the Bonferroni corrected Dunn’s test (SAS Institute, Cary, NC, USA; SAS Institute Inc. 2018). For the 2017 and 2018 paired host study, captures of *T. japonicus* were compared by tree species pair using the Wilcoxon signed-rank test. In 2019, captures were compared among the three host species using the Kruskal–Wallis test followed by the Bonferroni corrected Dunn’s test. All statistical comparisons used SAS 9.4 [30] and were considered significant at *p* < 0.05.

## 3. Results

### 3.1. Seasonal Captures of Trissolcus japonicus

**Figure 2:**
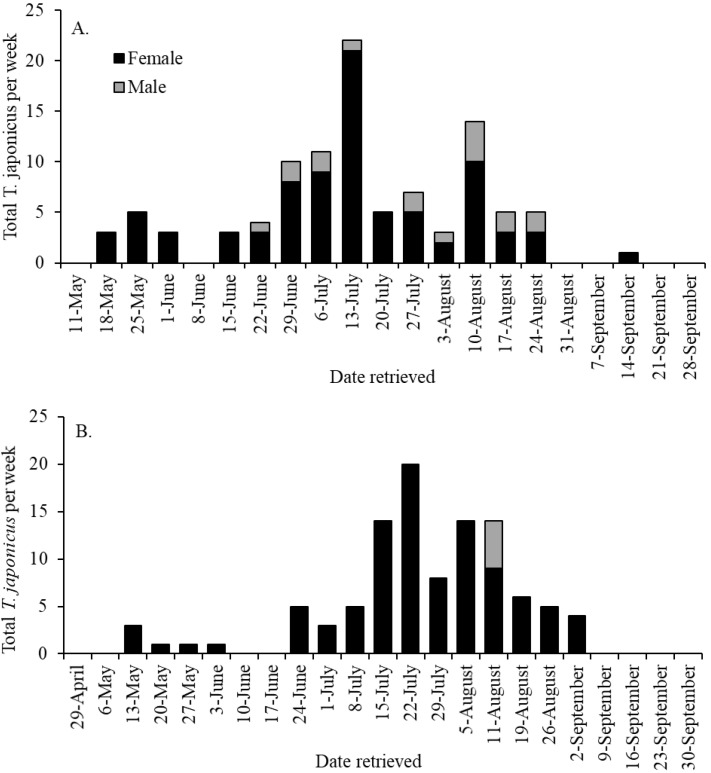
Weekly captures of *Trissolcus japonicus* in yellow sticky traps in the mid-canopy of mature female tree of heaven in Frederick and Shenandoah counties, Virginia, USA in (**A**) 2018 and (**B**) 2019. Captures in isolated patches, windbreaks, and woodlands were pooled by week.

In 2018 and 2019, respectively, 101 (83.2% female) and 104 (95.2% female) *T. japonicus* were captured across all habitats sampled (Table 1 and Table 2). In 2018, the first capture was recorded on 18 May (Figure 2A) and captures occurred on most weeks through 14 September, with peak captures on 13 July and 10 August. The last detection of *T. japonicus* was recorded on 14 September. Interestingly, males were captured only between mid-June and late August. In 2019, the date of first *T. japonicus* capture, 13 May (Figure 2B), was similar to that in 2018. Again, *T. japonicus* was captured on most weeks between mid-May and early September, but males were recorded only on 11 August. Additionally, similar to 2018, peak captures in 2019 occurred on 22 July and between 5 and 11 August, and the last capture was recorded on 2 September.

### 3.2. Effect of Habitat Type on T. japonicus Captures

**Figure 3:**
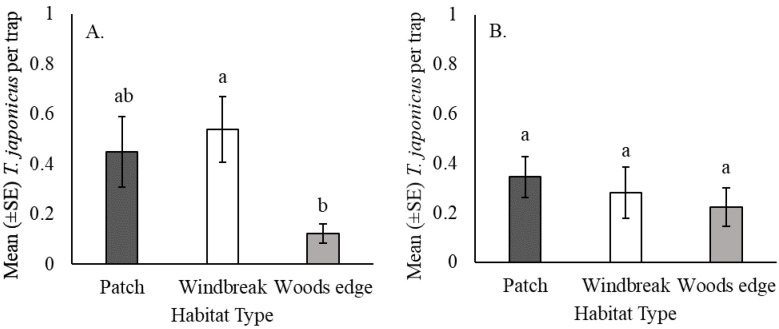
*Trissolcus japonicus* captures in yellow sticky traps in the mid-canopy of mature female tree of heaven in Frederick and Shenandoah counties, Virginia, USA by habitat type in (**A**) 2018 and (**B**) 2019. Bars with the same letter are not significantly different (Kruskal–Wallis test and Bonferroni corrected Dunn’s test, *p* < 0.05).

In 2018, there was a significant effect of habitat type on *T. japonicus* captures (χ^2^ = 8.31, df = 2, *p* < 0.05); significantly more were captured in windbreaks than at the woods edge (Z_windbreak_ = 7.9, Z_woods edge_ = 6.9, *p* < 0.05), with no difference between the woods edge and isolated patches (Z_woods edge_ = 6.9, Z_patch_= 7.4, *p* > 0.05) or between windbreaks and isolated patches (Z_windbreak_ = 7.9, Z_patch_ = 7.4, *p* > 0.05) (Figure 3A). In 2019, however, habitat type did not significantly affect captures (χ^2^ = 0.25, df = 2, *p* > 0.05) (Figure 3B).

### 3.3. Trissolcus japonicus Captures in Paired Host Trees at the Woods Edge

**Figure 4:**
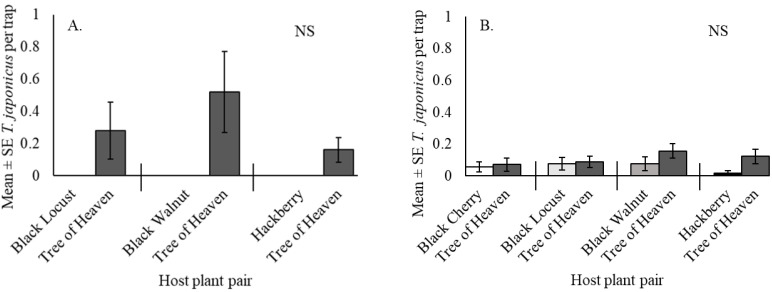
*Trissolcus japonicus* captures in yellow sticky traps in the mid-canopy of paired tree hosts of Halyomorpha halys (*n* = 5 per host pairing) at the forest edge in Frederick Co., VA, from (**A**) 31 July until 29 August 2017 and (**B**) 13 June until 20 September 2018. NS signifies no significant difference within host plant pairs at *p* < 0.05.

In 2017, 24 *T. japonicus* were captured during the four weeks of sampling in July and August (Table 3). All captures of *T. japonicus* were from tree of heaven, although captures did not differ significantly among the native tree species; tree of heaven vs black locust, S = −3, *p* > 0.05, tree of heaven vs black walnut, S = −5, *p* > 0.5, tree of heaven vs hackberry, S = −3, *p* > 0.05 (Figure 4A). 

In 2018, 42 *T. japonicus* were captured on traps during 12 weeks of sampling between June and September (Table 4). Of all *T. japonicus* captures, 66.7% were from tree of heaven, and captures among the native tree species did not differ significantly; tree of heaven vs black locust, S = −1.5, *p* > 0.05, tree of heaven vs black walnut, S = −10, *p* > 0.05, tree of heaven vs hackberry S = −11, *p* > 0.05, tree of heaven vs black cherry, S = −1, *p* > 0.05 (Figure 4B).

### 3.4. Trissolcus japonicus Captures Among Three Tree Species in Windbreaks

**Figure 5:**
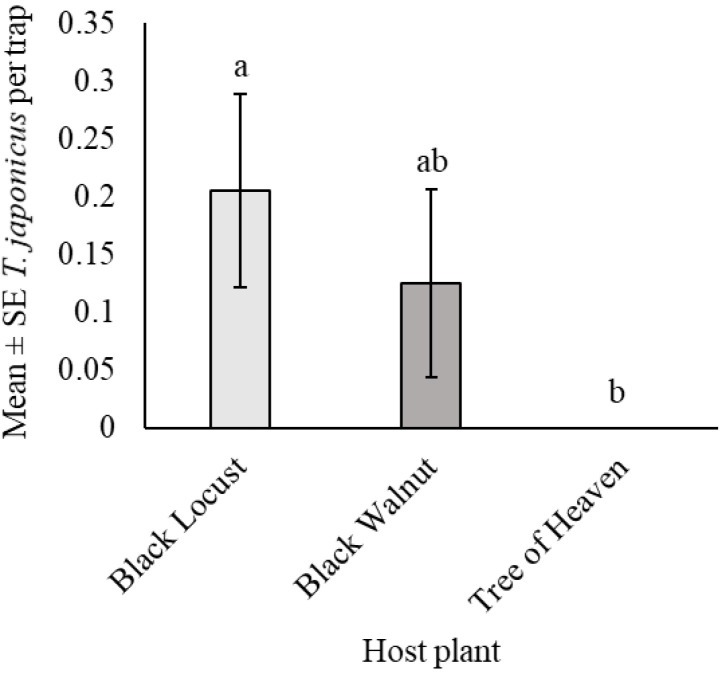
*Trissolcus japonicus* captures in yellow sticky traps in the mid-canopy of three tree hosts of *Halyomorpha halys trees* in the same windbreaks (*n* = 5 sites) in Frederick Co., VA, from 17 June until 11 August 2019. Bars with the same letter are not significantly different (Kruskal–Wallis test and Bonferroni corrected Dunn’s test, *p* > 0.05).

During the 8-week sampling period between June and August 2019, only 13 *T. japonicus* were captured (Table 5), 61.5, 38.5, and 0.0% of which were from black locust, black walnut, and tree of heaven, respectively. Captures of *T. japonicus* differed significantly among the tree species in which traps were deployed (χ^2^ = 6.32, df = 2, *p* < 0.05); significantly more were captured in black locust than in tree of heaven (z _black locust_ = 8.4, z _tree of heaven_ = 7.3, *p* < 0.05) (Figure 5), while captures in black walnut were not significantly different from those in black locust (z _black locust_ = 8.4, z _black walnut_ = 7.8, *p* > 0.05) or tree of heaven (z _black walnut_ = 7.8, z _tree of heaven_ = 7.3, *p* > 0.05). 

### 3.5. Captures of Native Parasitoids of Halyomorpha halys 

In the habitat type study, 61 and 159 specimens of native parasitoids of *H. halys* were captured across the habitats sampled in 2018 and 2019, respectively (Table 1 and Table 2). All of the native species captured and listed in Table 1 and Table 2 are known to develop successfully, to varying degrees, in *H. halys* sentinel eggs [4]. In 2018, these included *Trissolcus brochymenae* (Ashmead), *Trissolcus euschisti* (Ashmead), *Trissolcus thyantae* Ashmead, *Trissolcus edessae* Fouts, *Telenomus podisi* Ashmead, *Telenomus* spp., Anastatus spp., and Encyrtidae (Table 1). Captures in 2019 included *Trissolcus brochymenae*, *Trissolcus thyantae*, *Trissolcus euschisti, Telenomus podisi*, *Telenomus persimilis* Ashmead, *Telenomus* spp., *Gryon* spp. (Scelionidae), *Anastatus* spp., and Encyrtidae (Table 2). 

In the paired host study, 12 and 52 specimens of native Scelionidae were captured in 2017 and 2018, respectively (Table 3 and Table 4). In 2017, these included *Trissolcus brochymenae*, *Trissolcus euschisti*, *Telenomus* spp., and *Gryon* spp. (Table 3). Captures in 2018 included *Trissolcus thyantae*, *Trissolcus euschisti*, *Telenomus podisi* Ashmead, *Telenomus* spp., and *Gryon* spp. (Table 4). In 2019, 48 specimens of native scelionids were captured on traps in the three tree species in windbreaks, including *Trissolcus brochymenae*, *Trissolcus thyantae*, *Trissolcus euschisti*, *Trissolcus edessae*, *Telenonus podisi*, and *Telenomus* spp. (Table 5).

## 4. Discussion

Phenological synchrony of parasitoids with their hosts influences parasitoid population size and rate of colonization, with phenological mismatch potentially reducing observed parasitism of *H. halys* eggs by *T. japonicus* in Beijing, China [31]. Sampling in two consecutive years revealed first detections of *T. japonicus* in mid-May, peak captures in mid-July and early August, a marked decline in late August, and last detections in early to mid-September. The onset of captures in May coincided with the period of peak *H. halys* emergence from overwintering sites in the eastern USA [32] and peak captures followed predicted periods of peak *H. halys* oviposition [33]. As it does for other species [34], this synchrony should increase the likelihood of the persistence of adventive *T. japonicus*, supported by consistent indications of its range expansion in the USA in recent years [1]. Importantly, the seasonality of *T. japonicus* captures aligned well with the seasonal parasitism of *H. halys* eggs by *T. japonicus* in China [10], based on sentinel egg mass deployments at regular intervals from May or June through August or September. Moreover, declining detections in late August aligned with the cessation of *H. halys* oviposition by approximately mid-August [35], despite highest annual *H. halys* populations from late August through much of September [36,37]. While the overwintering biology of *T. japonicus* remains poorly understood, declining captures starting in late August may indicate that *T. japonicus* enters overwintering sites during this period. Similar total captures in 2018 and 2019 were notable given that 2019 was much drier than 2018 [38], suggesting that the abundance of *T. japonicus* remained stable despite annual climate variation, also boding well for its persistence. Our documentation of seasonal changes in captures of adventive *T. japonicus* in the Mid-Atlantic USA can inform the timing and efficiency of *T. japonicus* surveillance in *H. halys* host trees, particularly given the concurrence with results from China [10].

It is generally believed that decreased habitat size and increased fragmentation reduce parasitoid abundance [39] and parasitism [40]. Thus, highest captures of *T. japonicus* might have been expected in our largest and most contiguous habitat type, woods edge. However, in 2018, the fewest *T. japonicus* were captured at the edge of woodlots and most were captured in windbreaks, with no significant differences in captures among all habitats in 2019. These findings suggest site-specific variability in *T. japonicus* abundance. Positive density-dependent responses of parasitoids to hosts have been documented for other *Trissolcus* species [41,42], and *T. japonicus* abundance was likely influenced by *H. halys* density. While we did not monitor *H. halys* populations at the study sites, simultaneous sampling of *H. halys* and *T. japonicus* in future research may prove instructive. 

Given the broad host range of *H. halys* [27] and the diversity of feeding and reproductive hosts available in this region [26], in theory, *T. japonicus* foraging for egg masses should not be limited by tree species. Rather, *T. japonicus* foraging may be most strongly associated with the presence or density of *H. halys* and its egg masses. Counts of *H. halys* egg masses on ornamental trees in an urban landscape by Formella et al. [43] revealed no significant differences among hosts in egg mass numbers, confirming *H. halys* oviposition on many plant species. However, their ground-based counts via visual observations may have underestimated egg mass density, based on data [20] showing greater numbers of *H. halys* egg masses producing *T. japonicus* from those collected at mid-canopy compared with other tree strata. In ornamental tree nurseries, more *H. halys* egg masses were found on angiosperms than gymnosperms, spanning numerous plant species and families [44]. Boyle et al. [45] suggested that *T. japonicus* may respond primarily to kairomones left by gravid *H. halys* walking on plant surfaces. If the distribution of *H. halys* populations are stochastic and show spatial and temporal changes in relative density, the distribution of *T. japonicus* might be expected to show the same trend, thereby resulting in changing parasitoid densities in a given area over time [46]. The curious differences in *T. japonicus* captures between native hosts and tree of heaven in 2017–2018 and 2019 may reflect this stochastic process. 

Our studies using tree of heaven for standardized sampling have shown it to be a productive species for detecting *T. japonicus*. Across several studies in 2020, > 500 *T. japonicus* were captured in yellow sticky traps in female tree of heaven (Dyer, unpublished data). However, these data indicate that surveillance for *T. japonicus* need not be limited to specific *H. halys* host trees, thus enabling greater sampling flexibility. In other parts of the USA, sentinel *H. halys* eggs yielded *T. japonicus* detections from vine maple (*Acer circinatum*), *Catalpa* sp. [23], and English holly (*Ilex aquifolium*) [12].

Native parasitoids that attack *H. halys* eggs were captured, and in most studies, captures of *T. japonicus* were much higher than those of any other species. The community of native parasitoids observed was similar in composition to that reported by Tillman [47] in GA, USA, where *T. japonicus* has not yet been detected. Additionally, *T. japonicus* attacked more *H. halys* eggs than native stink bug eggs in field choice trials [23], suggesting that, as Konopka et al. [48] concluded, *T. japonicus* may be able to successfully coexist with native species in the biological control of *H. halys*. However, long-term effects of the addition of *T. japonicus* to the community of pentatomid parasitoids remain to be determined and warrant continued monitoring. Longitudinal studies using sticky traps alone or in concert with or other sampling methods, such as sentinel egg masses, may provide an indication of the impact of *T. japonicus* on the relative abundance of native *H. halys* parasitoids. 

## 5. Conclusions

These studies further validate previous results [21] by showing that yellow sticky traps were effective for *T. japonicus* monitoring and surveillance. Detections of *T. japonicus* in several different habitats and BMSB host tree species indicated flexibility in the spatial component of *T. japonicus* sampling. Temporally, the consistently greatest captures from mid-June through early August can inform the timing of sampling and increase the likelihood of its detection. As discussed previously [21], yellow sticky traps are effective for addressing questions about the presence of *T. japonicus*, communities of native *H. halys* parasitoids, and the spread of *T. japonicus* in the invaded range of *H. halys,* but do not replace the use of sentinel or wild egg masses to assess *H. halys* egg parasitism or the impacts of parasitism on its populations.

## Figures and Tables

**Table 1:** Adventive and native parasitoids of *Halyomorpha halys* captured in yellow sticky traps in the mid-canopy of mature female tree of heaven in Frederick and Shenandoah counties, Virginia, USA 2018.

Habitat	*Trissolcus*					*Telenomus*		*Anastatus*	Encyrtidae
	*japonicus*	*brochymenae*	*thyantae*	*euschisti*	*edessae*	*podisi*	spp.	spp.	
Patch	40	2	0	4	0	2	2	2	0
Windbreak	50	6	0	14	1	3	2	1	0
Woods edge	11	2	1	11	0	4	5	4	2
Total	101	10	1	29	1	9	9	7	2

**Table 2:** Adventive and native parasitoids of *Halyomorpha halys* captured in yellow sticky traps in the mid-canopy of mature female tree of heaven in Frederick and Shenandoah counties, Virginia, USA 2019.

Habitat	*Trissolcus*				*Telenomus*			*Gryon*	*Anastatus*	Encyrtidae
	*japonicus*	*brochymenae*	*thyantae*	*euschisti*	*podisi*	*persimilis*	spp.	spp.	spp.	
Patch	43	2	5	18	20	0	9	1	5	2
Windbreak	35	4	7	25	17	1	2	1	5	0
Woods edge	26	4	7	15	6	2	10	0	4	1
Total	104	10	19	58	43	3	21	2	14	3

**Table 3:** Adventive and native parasitoids of *Halyomorpha halys* captured in yellow sticky traps in the mid-canopy of paired *H. halys* host trees (*n* = 5 per host pairing) at the forest edge in Frederick Co., VA, from 31 July until 29 August 2017.

Host Plant	*Trissolcus*			*Telenomus*	*Gryon*
	*japonicus*	*brochymenae*	*euschisti*	spp.	spp.
Black Locust	0	1	1	3	1
Tree of Heaven	7	1	1	0	0
Black Walnut	0	0	0	0	0
Tree of Heaven	13	1	1	0	0
Hackberry	0	0	0	0	0
Tree of Heaven	4	0	2	0	0
Total	24	3	5	3	1

**Table 4:** Adventive and native parasitoids of *Halyomorpha halys* captured in yellow sticky traps in the mid-canopy of paired *H. halys* host trees (*n* = 5 per host pairing) at the forest edge in Frederick Co., VA, from 13 June until 20 September 2018.

Host Plant Pair	*Trissolcus*			*Telenomus*		*Gryon*
	*japonicus*	*thyantae*	*euschisti*	*podisi*	spp.	spp.
Black Cherry	3	1	6	1	1	0
Tree of Heaven	4	0	1	0	0	0
Black Locust	5	0	4	1	1	0
Tree of Heaven	6	0	3	6	0	0
Black Walnut	5	0	3	0	0	0
Tree of Heaven	10	0	5	0	0	0
Hackberry	1	1	5	2	0	1
Tree of Heaven	8	0	8	1	1	0
Total	42	2	35	11	3	1

**Table 5:** Adventive and native parasitoids of *Halyomorpha halys* captured in yellow sticky traps in the mid-canopy of *H. halys* host trees in windbreaks (*n* = 5 sites) in Frederick Co., VA, from 17 June until 11 August 2019.

Host Plant	*Trissolcus*					*Telenomus*	
	*japonicus*	*brochymenae*	*thyantae*	*euschisti*	*edessae*	*podisi*	spp.
Tree of Heaven	0	1	2	6	1	9	2
Black Locust	8	1	0	9	0	2	0
Black Walnut	5	1	0	6	0	6	2
Total	13	3	2	21	1	17	4

## Data Availability

Data available upon request.
